# Aerial surveys cause large but ephemeral decreases in bear presence at salmon streams in Kodiak, Alaska

**DOI:** 10.1371/journal.pone.0222085

**Published:** 2019-09-10

**Authors:** William W. Deacy, William B. Leacock, Eric J. Ward, Jonathan B. Armstrong

**Affiliations:** 1 Department of Fisheries and Wildlife, Oregon State University, Corvallis, OR, United States of America; 2 Arctic National Wildlife Refuge, United States Fish and Wildlife Service, Kodiak, AK, United States of America; 3 Conservation Biology Division, Northwest Fisheries Science Center, National Marine Fisheries Service, National Oceanic and Atmospheric Administration, Seattle, WA, United States of America; Université de Sherbrooke, CANADA

## Abstract

Aerial surveys are often used to monitor wildlife and fish populations, but rarely are the effects on animal behavior documented. For over 30 years, the Kodiak National Wildlife Refuge has conducted low-altitude aerial surveys to assess Kodiak brown bear (*Ursus arctos middendorffi*) space use and demographic composition when bears are seasonally congregated near salmon spawning streams in southwestern Kodiak Island, Alaska. Salmon (*Oncorhynchus spp*.) are an important bear food and salmon runs are brief, so decreases in time spent fishing for salmon may reduce salmon consumption by bears. The goal of this study was to apply different and complementary field methods to evaluate the response of bears to these aerial surveys. Ground-based counts at one stream indicated 62% of bears departed the 200m-wide survey zone in response to aerial surveys, but bear counts returned to pre-survey abundance after only three hours. Although this effect was brief, survey flights occurred during the hours of peak daily bear activity (morning and evening), so the three-hour disruption appeared to result in a 25% decline in cumulative daily detections by 38 time-lapse cameras deployed along 10 salmon streams. Bear responses varied by sex—male bears were much more likely than female bears (with or without cubs) to depart streams and female bears with GPS collars did not move from streams following surveys. Although bears displaced by aerial surveys may consume fewer salmon, the actual effect on their fitness depends on whether they compensate by foraging at other times or by switching to other nutritious resources. Data from complementary sources allows managers to more robustly understand the impacts of surveys and whether their benefits are justified. Similar assessments should be made on alternative techniques such as Unmanned Aerial Vehicles and non-invasive sampling to determine whether they supply equivalent data while limiting bear disturbance.

## Introduction

Wildlife researchers often use aerial surveys to collect data on animal distributions, abundance, demography, and behavior. Aerial surveys are a particularly valuable tool in rugged, vast, and remote study areas where other methods of observing animals are difficult or too expensive. Although historically, surveys have been conducted by observers in fixed-winged aircraft and helicopters, Unmanned Aerial Vehicles (UAVs or drones) are becoming increasingly common as their capabilities improve and costs decrease [1,2]. Regardless of the platform, researchers have long recognized that aerial observation can disturb target and non-target wildlife. For example, mountain goats (*Oreamnos americanus*) [3], caribou (*Rangifer tarandus*) [4], Mexican spotted owls (*Strix occidentalis*) [5], feral horses (*Equus ferus caballus*) [6], and brown bears (*Ursus arctos*) [7] have all been observed to alter their behavior in response to aerial surveys. Responses range from relatively benign (brief increase in alertness) to substantial (rapid long-distance movements) and vary across species and even populations in proportion to the risk perceived by the animal [8].

Prior studies have used a variety of methods to detect behavior changes caused by survey aircrafts or UAVs. Several studies have used ground observations of animal behavior and movements in response to real [7,9–11] or loudspeaker-simulated survey flights [5,12]. Others have implanted heart rate monitors into animals to document physiological as well as behavioral responses to aircraft overflights [12,13]. It is clear that animals may respond in a manner that is detectible using one method, but not others [13], and some methods (like short-term observations) are unable to document the duration of behavioral changes. To get a complete picture of the magnitude and duration of the effect of flights on wildlife, it is advantageous to measure and analyze multiple complementary datasets that vary in spatial and temporal scale and can distinguish among demographic groups within a population.

Since 1982, the Kodiak National Wildlife Refuge, Alaska (KNWR), has conducted systematic aerial Brown Bear Stream Surveys (BBSS) to monitor Kodiak brown bear space use and demographic composition where bears seasonally congregate to forage on salmon along a suite of 6–11 rivers and streams [14]. Spawning salmon are an important food resource for bears in this area, accounting for 48% and 68% of average assimilated diets for females and males, respectively [15]. Bears aggregate at high densities (>20 individuals/ stream km; WBL *unpublished data*) when salmon are abundant. The BBSS was designed to detect changes in bear use of salmon streams and demographic composition, such as the ratio of single bears to family groups [14]. Demographic shifts can signal a decline in the population growth rate [16]. Aerial surveys are an effective tool for monitoring bears in southwest Kodiak because it is very remote (there is no road access) and covers a broad area (~1200 km^2^). The BBSS survey protocol consists of three flight replicates (intended to be approximately 12 hours apart) per week starting in early July and ending the third week of August. Given suitable weather, the surveys are flown slowly (~70 knots) and at low altitude (~120–150 meters above ground level) to maximize bear detection rates (more details in Methods).

In response to concerns that the BBSS was displacing bears away from salmon streams, the Kodiak National Wildlife Refuge initiated this study. It was not our objective to assess the rationale or efficacy of the BBSS, but rather to determine whether the aerial surveys caused bears to leave the area around salmon spawning streams. If bear presence did decrease, we wanted to quantify for how long and whether they rebounded to original levels. The population we studied is in a remote part of Kodiak Island, where there are few other sources of anthropogenic disturbance except for fishing, recreational sport hunting for deer, bears, and mountain goats, isolated bear-viewing operations, and research activities. None of these activities have the same temporal or spatial extent as the aerial surveys. Due to past studies which found that bears responded to low-flying aircraft [7], we hypothesized that bear use of salmon streams would decrease during and following surveys. However, based on observations that bear abundance is often high during the aerial monitoring period, we hypothesized bears were not permanently leaving stream sites and would return to pre-survey levels rapidly. We used four complementary sources of data to test our hypotheses, that each have strengths and weaknesses (Table 1). Bear counts from the aerial surveys themselves provided long-term, temporally coarse data at regional spatial extent. Ground-based spotting scope surveys of one of the streams (Connecticut Creek) provided counts at higher temporal resolution (but lower spatial and temporal extent) and included demographic covariates (i.e., sex and cub status). Time-lapse cameras (n = 38 cameras) distributed along ten survey streams provided a spatially and temporally extensive dataset that allowed us to estimate the cumulative effect of aerial surveys over several days but neither captured the response of individual bears nor provided reliable estimates of short-term bear responses. Finally, GPS locations from collared female bears (n = 52), provided individual-level responses to aerial surveys and allowed us to quantify the spatial magnitude of displacement for female bears only. We predicted that aerial and ground-based survey counts, and time-lapse camera detections of bears, would decrease following survey flights. We predicted bears fitted with GPS collars would move further away from streams following aerial survey flights. We predicted all metrics of bear use of salmon streams to return to pre-survey levels within 24 hours (a span of time that encompasses and thus accounts for the considerable diel variation in bear activity).

**Table 1:** Survey/data types and characteristics.

Survey Type	Measure	Spatial Extent	Spatial Grain	Temporal Extent	Temporal Grain	Demographic Resolution	Type of Information Provided
**Aerial Survey Counts**	Counts of bears within 100m of streams	1982–2005: 6 sites 2006–2007: 0 sites 2008–2012: 11 sites 2013: 14 sites 2014–2015: 11 sites	Counts of bears observed within 100m of streams were summed by stream.	Approximately 7/10–8/31 from 1982–2015 (n = 31 years).	During 2–4 day survey periods, survey reps occurred every 12.8 (+-3.4) hours	Family groups and single bears.	Change in number of bears between survey reps.
**Ground Surveys**	Counts of bears within 100m of streams	One site (Connecticut Creek)	Counts of bears observed within 100m of the stream were summed.	600 surveys over 28 days. July and August of 2014 (n = 266), and 2015 (n = 334).	Survey scans occurred every 15 minutes	Family groups, single bears, males, females.	Short term response of bears. Duration of response.
**GPS Collars**	Distance from aerial survey streams	2008–2012: 11 sites 2013: 14 sites 2014–2015: 11 sites	Mean GPS errors were <20m [17]	All aerial surveys from 2008–2011 and 2014–2015	Hourly GPS fix frequency.	Only females (n = 52).	Change in space use following survey flights.
**Time-Lapse Cameras**	Bear detections	10 streams/rivers across approximately 1000km^2^.	At least 3 cameras spaced along the length of each stream/river	June–September, 2013–2015	Images every 5 minutes, but detections must be aggregated by day for coherent variation	Family groups and single bears.	Cumulative time-integrated effects of series of multiple aerial surveys

## Methods

### Study area

**Fig 1:**
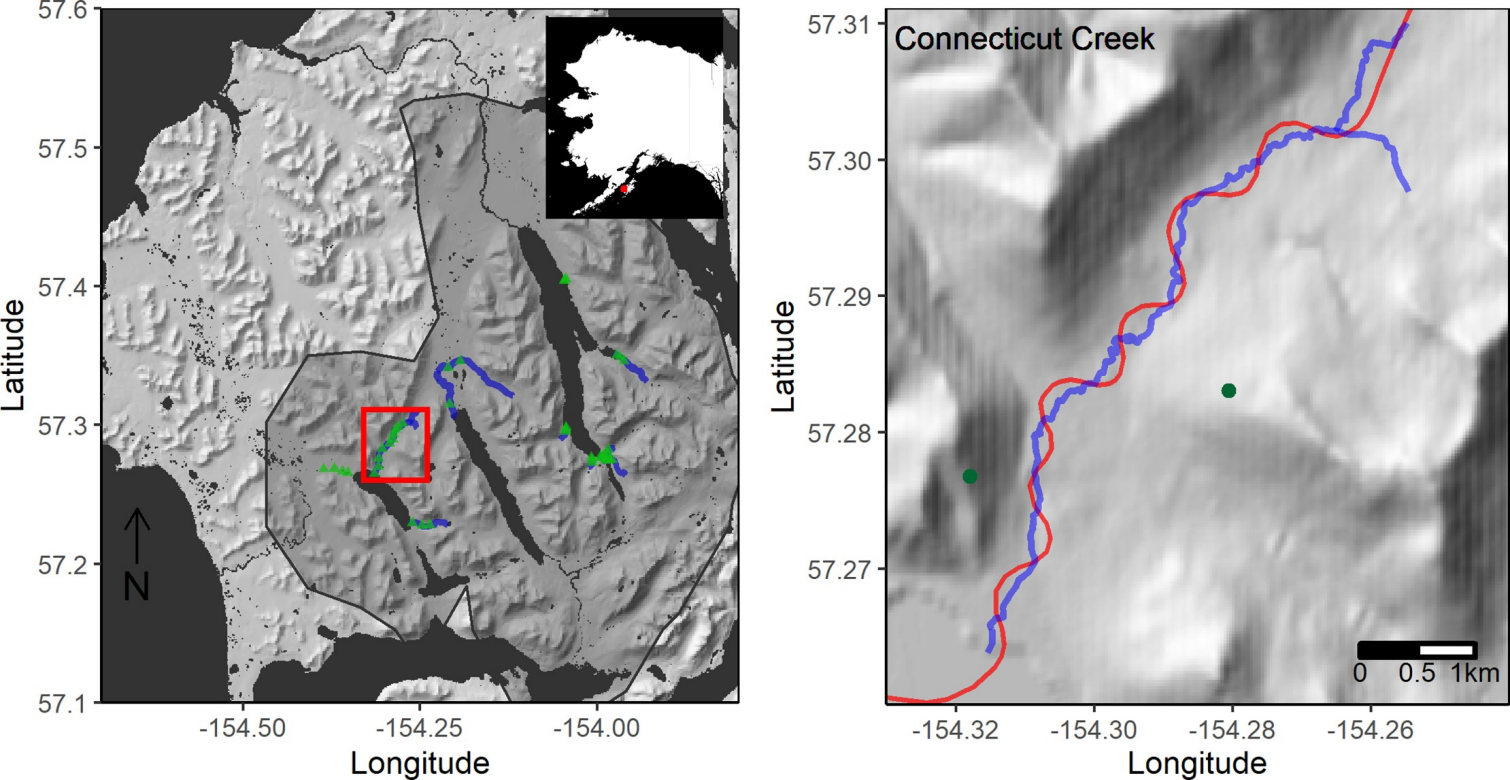
Study area map. Map of Kodiak, Alaska study area (left) with streams/rivers where aerial surveys occurred in blue. Green triangles show the locations of time lapse cameras, and the grey polygon shows a 90% utilization distribution of female brown bear GPS locations with a smoothing factor (h) of 0.04. The right panel shows Connecticut Creek with the GPS track of a typical aerial survey flight in red and the locations of the hill camps where spotting-scope surveys occurred as green points. Basemaps were created using publicly available data from the US Geologic Survey.

Fieldwork was conducted from 1982–2015 in the southwestern portion of Kodiak Island, Alaska, an approximately 1,000 km^2^ area with three primary salmon nursery lakes (Karluk, Frazer, and Red), dozens of spawning tributaries, and a dense population of brown bears (~ 250 individuals/1000 km^2^) [18]. The study area (see [19] for description) includes dozens of streams and rivers that are currently or were historically important habitats for bears to fish for salmon (Fig 1). Permission to conduct fieldwork was granted by the Refuge Manager for the Kodiak National Wildlife Refuge.

### Description of brown bear stream survey flights

The Brown Bear Stream Surveys were flown in small tandem two-seat aircraft (Aviat Husky or CubCrafters Top Cub), with both pilot and passenger observing. All bears spotted within 100 meters of the respective streams were counted. The backseat observer recorded data, classifying bear observations as single bears, sows with cubs of the year (COY), or sows with older cubs. Weather permitting, three low level (~120–150 meters above ground level), slow flying (~70 knots) surveys were conducted per week, with early morning (0700) or late afternoon (1800) start times. Weather permitting (without fog or high-winds), sequential surveys followed a morning-evening-morning or evening-morning-evening sequence. The sequence by which streams were surveyed was alternated on successive survey repetitions to limit bias caused by diel changes in bears at streams (Figure A in S1 File). From 1982–2015, the following streams/rivers were surveyed: Connecticut Creek, Southeast Creek, Red Lake River, Pinnell Creek, Sturgeon River, and the East Sturgeon River. In addition, Thumb River, O’Malley River, Moraine, Creek, Meadow Creek, and the Dog Salmon River were flown from 2008–2015 and Halfway Creek, Grassy Creek, and Cottonwood Creek were flown in 2013. No surveys were flown from 2006–2007 (Table 1).

### Aerial counts on successive surveys

To test whether surveys decreased bear presence near salmon streams (defined for aerial surveys as the area within 100m of the stream), we compared sequential aerial survey bear counts that occurred less than 24 hours apart (follow-up flights occurred an average of 12.8 +/- 3.4 hours after prior flights). If aerial surveys were displacing bears from the survey area, later repetitions in a sequence of surveys should have lower bear counts. We used a generalized linear mixed model (using the package *glmmTMB* in *R* [20]) with survey sequence number and time of day (AM or PM) as fixed effects, random intercepts for year and stream, and a poisson error structure (log link function). Next, we fit a linear mixed effects model (using the package *lmer* in *R* [21]) to test whether time since the previous aerial survey was a significant predictor of the difference in number of bears counted. Our response variable was the bear count in the first of two surveys minus the bear count in the second survey. The model included time of day (AM or PM) and hours since previous survey as fixed effects, random intercepts for year and stream, and a gaussian error distribution (identity link function). We only included replicate surveys that had occurred within 24 hours of the prior survey (*n* = 116), which likely reduced confounding variation in salmon abundance in streams, berry abundance away from streams, or weather conditions, as these do not vary as strongly at diel timescales as they do at daily to weekly extents.

### Ground based bear counts

Past research in the study area found that bear counts from concurrent aerial and ground surveys largely agreed [22]. We conducted similar ground surveys from one of two hilltop locations near Connecticut Creek (Fig 1) in 2014 and 2015. The observation points allowed observers to see almost all of Connecticut Creek. They were approached without walking through the aerial survey area. Scans of all bears occurred every 15 or 30 minutes, depending on the number of bears (if there were many bears, the survey exceeded 15 minutes). Bears were counted and classified by demographic class (adult of unknown sex, adult female, adult male, sow with cubs, subadult male, subadult female, and subadult of unknown sex). These classifications can occasionally be incorrect [23], but surveyors were instructed to only classify when their confidence was high. When surveyors were not confident, sex/class was not recorded, and these observations were excluded from sex-specific comparisons. Survey timing varied due to fog, but generally occurred from 6 AM to 10 AM and 6 PM to 10 PM. These time blocks were chosen because continuous surveying was not logistically feasible, and these are the periods when bears were most numerous and most active (Figure A in S1 File). These survey periods also encompassed the times when aerial surveys occurred.

The number of bears on streams commonly varies greatly through time, so each sequence of hill camp observations was standardized by dividing by the maximum value in each sequence. For example, if a sequence of 30-minute frequency surveys beginning at 6AM and continuing until 10AM had values {2, 1, 4, 2, 4, 0, 3, 2, 0} the standardized values would be {0.5, 0.25, 1, 0.5, 1, 0, 0.75, 0.5, 0}. Next, to allow use of a beta distribution, response values were transformed so values of 1 were changed to 0.9999999 and values of 0 were changed to 0.0000001. The raw data can be seen in the supplement (Figure B in S1 File). To model variability in counts of bears from the hill camps before and after aerial survey flights, we fit a global generalized linear mixed model using glmmTMB [20]. Using the standardized data y*_d,*t*_ as the response, the form of the model was y*_d,*t*_ *= a*_*d*,*t*_*+s*_*d*,*t*_*+r*_*d*,*t*_*+z*_*d*,*t*_ *+a*_*d*,*t*_:*s*_*d*,*t*_:*r*_*d*,*t*_, where *a*_*d*,*t*_ is an indicator of whether the count is before (0) or after (1) the flight (or average flight time on non-flight days), *s*_*d*,*t*_ is an indicator of whether (1) or not (0) the observation occurred during a flight day, *r*_*d*,*t*_ is the number of hours from the survey time (negative if before the survey, positive after the survey, and *z*_*d*,*t*_ is a factor indicating the time of day (morning or evening). The model was fit with a beta error distribution (logit link function). Because the hill camp counts within each sequence were not independent, and the standardizing procedure described above does not reduce temporal autocorrelation among counts, we fit a model with a first-order autoregressive covariance structure (AR1). This covariance assumes data points spaced closer in time are more correlated than points separated by greater time lags, $cov(y_{d,t}^{*},y_{d+x,t}^{*})=\sigma^{2}exp(-\theta |x|)$, where *exp*(−*θ*) is equivalent to the correlation between adjacent counts. To see how results differed by bear class, we fit additional global models (with the same form used with all bears) focused on counts of bear families (a sow with one or more cubs counted as one family), and male bears. There was not enough variation in the response variable for single female bears (because of too few observations, *n* = 63), so they were excluded from the class-specific analysis.

For each bear class (all independent bears, family groups, and males), we fit all combinations of fixed effects provided by the global model, including interactions, using dredge (in package MuMin [24]). We selected among the resulting models based on AICc [25] to arrive at top models for each bear demographic.

### Time-lapse camera data

From 2013–2015, time-lapse cameras were deployed along ten streams within aerial survey zones (Fig 1). Streams included: Connecticut Creek, Southeast Creek, Red Lake River (2015 only), Pinnell Creek, Meadow Creek, O’Malley River, Falls Creek, Canyon Creek, Cascade Creek, and Upper Thumb River. Each stream had 3 cameras deployed during the salmon spawning season, except for Connecticut Creek, which had 10 cameras deployed in 2015, and Red Lake River, which had 4 deployed in 2015. We distributed the cameras (Reconyx RC55/PC800, Holmen, WI or Day 6 Outdoors Plotwatcher Pro, Columbus, GA) evenly from the mouth to a point where we did not observe salmon during aerial surveys and found no evidence of past spawning during ground surveys (e.g., jaws or gill plates). Cameras were deployed in June before the salmon run began and were removed in September, after spawning concluded. Cameras were programmed to take a photo every five minutes during daylight hours. We counted the number of bears (excluding cubs) in each photo and then summed counts by hour across all cameras/sites. Only a fraction of the watershed is observed by cameras and most photos record no bears, so time-lapse detections only reliably index bear fishing activity when detections are aggregated across longer time periods (e.g. hours and days). Aggregated detection counts do not census bears, but rather, index the number of bears using salmon streams and have been corroborated by more direct monitoring techniques, such as GPS telemetry [19]. Although bears occasionally use streams for purposes other than foraging on salmon (e.g. travel corridor or water source) a prior study found that bears were detected by time-lapse cameras 61-times more frequently on streams when salmon were present compared to when salmon were absent [19]. Thus, we feel confident in using time-lapse detections as a proxy for fishing activity.

Bears have been known to avoid salmon streams due to human presence [26,27]. To minimize the chance of displacing bears, our cameras were serviced (data cards exchanged) every 10–21 days, mostly during the midday hours (1100–1600) when bear activity along streams tends to be low (Figure A in S1 File). The timing of these visits was not synchronized with aerial survey flights, occurring both well before and after flights. Because of this, any displacement of bears caused by camera servicing is unlikely to bias our analysis.

Evaluation of hourly-aggregated time-lapse camera bear data revealed strong seasonal [28] and diel (Figure A in S1 File) patterns in bear presence at streams. Seasonal changes in bear presence are likely due to changing resource abundance (salmon and other food resources), while diel patterns are typical for brown bears, which generally exhibit crepuscular activity patterns [29–31]. Because the aerial surveys did not occur at random times, analyses of bear detection data could be biased by the seasonal and diel patterns of bear presence. To prevent this potential source of bias, we decomposed the data into seasonal, diel and remaining variation using the seasonal decomposition of time series by locally weighted regression (LOESS) procedure, which is provided by the R [32] function *stl* [33]. This method fits LOESS lines to each time series (a year of data), that are tuned to remove different frequencies of variation and is commonly used to decompose continuous time series with high and low frequency variation [34]. We used LOESS fits with windows of 25 hours and 401 hours to remove diel and seasonal variation, respectively. The remainder, which we used in the compositing analysis, was calculated by subtracting the seasonal trend time series and the diel variation time series from the time series of raw bear detections.

We used compositing to examine the number of bears detected by time-lapse cameras before, during, and after aerial surveys. Compositing is appropriate in this case because bear numbers change for many unobserved reasons, and compositing allows testing for a signal within this noise [34]. Compositing involves selecting bear count data that falls within a certain number of days of aerial surveys (within 2 days before and 3 days after) and conducting tests to see whether bear counts changed on the day of flights. This six-day window is the longest window we could use without overlapping other clusters of aerial surveys. The statistical test uses bootstrapping, where we randomly select 10,000 six-day chunks of data (with replacement) that that did not have aerial surveys and test whether our aerial survey influenced observations were more extreme than these random composites. We conducted this test on data that was detrended using the method described above and centered on d = -1 (the day before the survey), meaning that the mean value at d = -1 was subtracted from the other mean values.

### Location data from GPS collars

From 2008 to 2015, 52 female brown bears were captured and collared with GPS collars as described in Deacy et al. [19]. All capture procedures were performed in accordance with guidelines and regulations approved by the Fish and Wildlife Service Institutional Animal Care and Use Committee (IACUC permit # 2012008, 2015–001). GPS collars recorded positions every hour. We screened GPS locations for accuracy, removing relocations with a positional dilution of precision (PDOP) greater than 10 [17]. To test whether bears moved away from streams in response to aerial surveys, we identified series of GPS locations that began 24 hours prior to aerial surveys and ended 24 hours after aerial surveys. To exclude series of locations that were not near aerial survey flight paths, we only included series that had at least one location within 50 m of streams during the 48-hour series of locations. Some collars suffered from poor fix rates, so we only included 213 series of points which had at least 30 locations. The resulting fix rate across all included GPS location series was 82%. We included data from all years, even though there were no coincident ground surveys and time-lapse cameras deployed from 2008–2012. We used distance from streams as a metric of whether bears were displaced by survey aircraft.

To model variability in distance from streams before and after aerial survey flights, we first centered the data by subtracting the distance just prior to each flight, y*_t_ *= y*_*t*_ *-y*_*t = 0*_, where *t* is the time (in hours) relative to the flight. Using the centered data y*_t_ as the response, we fit a generalized linear mixed model in glmmTMB [20] with a gaussian error structure (identity link function). The form of the model was y*_t_ *= a*_*t*_*+u*_*i*_, where *a*_*t*_ is an indicator of whether the response is before (0) or after (1) the flight and *u*_*i*_ is a random intercept for individual bear. Because the distances within each sequence of locations were not independent, we fit a model with a first-order autoregressive (AR1) covariance structure. This covariance assumes that data points spaced closer in time are more correlated than points separated by greater time lags, $cov(y_{t}^{*},y_{x,t}^{*})=\sigma^{2}exp(-\theta |x|)$, where *exp*(−*θ*) is equivalent to the correlation between adjacent distances.

## Results

### Aerial counts on successive surveys

**Fig 2:**
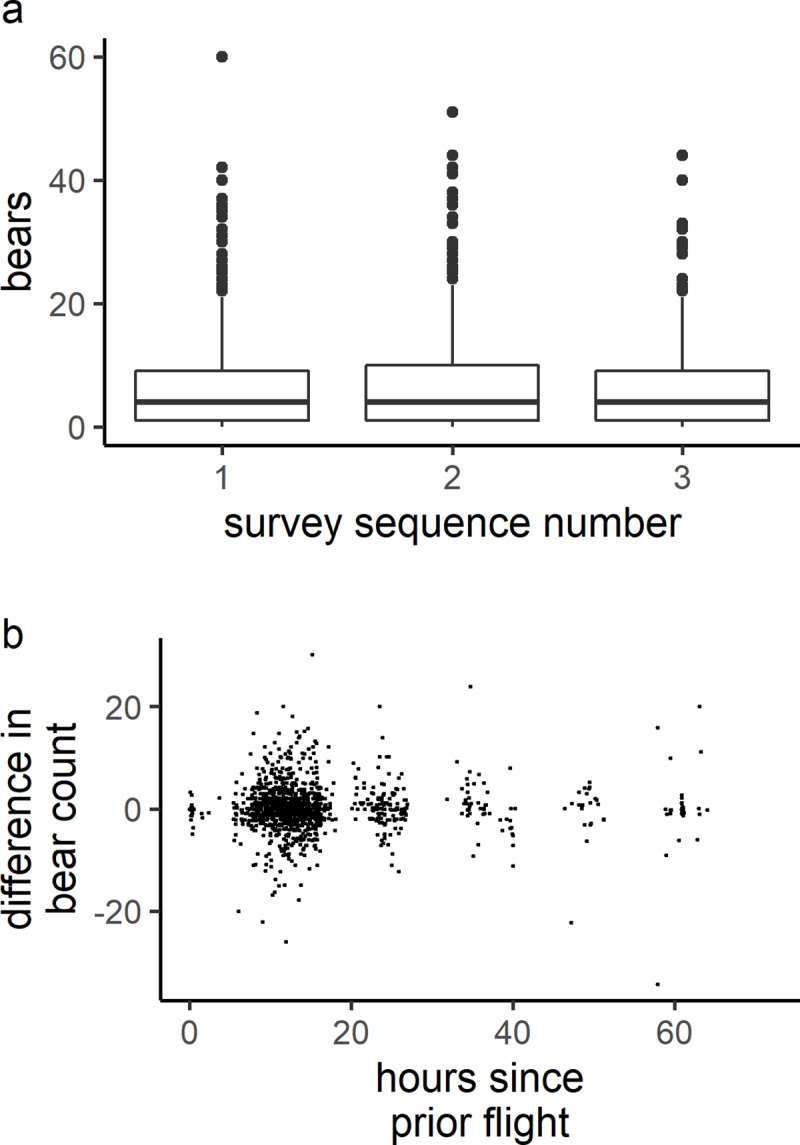
Comparison of bear counts from sequential aerial surveys occurring within 24 hours. a) count sequences (*n =* 106) showing the total bear count from aerial surveys. There was a modest average increase of 0.029 independent bears (2% increase) in each additional survey in a survey sequence (p = 0.03). b) plot showing the relationship between bear counts and time since the previous survey (*n =* 106). The effect of time since previous surveys on the number of observed bears was not significant at α = 0.05 (p = 0.61).

Of the 325 survey flights conducted since 1985, 106 occurred within 24 hours of a prior survey flight. Survey repetition was a statistically significant predictor (p = 0.03, α = 0.05) of bear count, however, the effect was small and in the opposite direction expected (Table A in S1 File); on average, surveyors observed 0.029 *more* independent bears (all bears minus cubs) in each additional survey in a survey sequence (an average increase of 2.0%) (Fig 2A). In addition, a linear mixed effects model showed that neither elapsed hours since the previous survey (p = 0.60) nor time of day (p = 0.21) were statistically significant predictors (α = 0.05) of the difference in number of bears observed during two sequential surveys (Fig 2B, Table B in S1 File).

### Ground based bear counts

**Fig 3:**
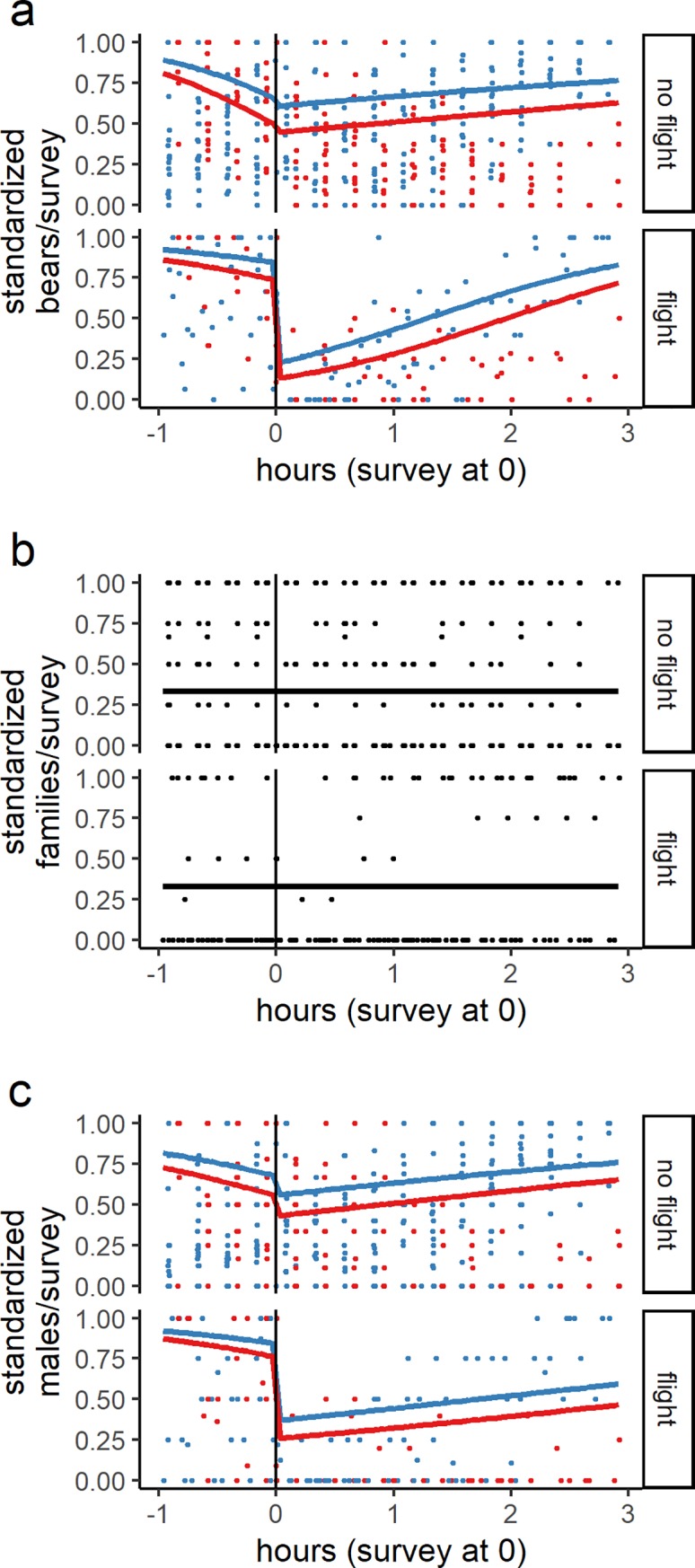
Bear responses as quantified by ground observations at Connecticut Creek, Alaska. Data has been divided between periods with aerial surveys (labeled flights) and no aerial surveys (labeled no flight). Points are jittered to alleviate over plotting. The x-axis shows the time relative to when surveys occurred (on flight days), or relative to the average time of flights on non-flight days. Lines show predicted values from top models for each bear class, with red points/lines indicating responses during the day and blue points/lines indicating responses during the evening. The number of bears varies greatly through time, so each sequence of hill camp observations was standardized to a range of 0 to 1 by dividing each observation by the maximum value in its series. a) all bears; b) family groups (a sow with cubs is counted as one); c) male bears. The male category only included observations where the observers were confident of the bear sex. The un-standardized raw data is displayed in Figure B in S1 File.

We conducted 55 series of spotting scope surveys (622 counts in total) from two hills above Connecticut Creek (Fig 1). Of these, 16 series were concurrent with aerial survey flights and 39 were not (Fig 3). Results from the model dredging process (details in Table C in S1 File) for independent bears (*n* = 3116 detections) resulted in a model with main effects for a binary before/after survey variable, time from survey flight, and binary flight day indicator variable. The top model also had the following two-way interactions: before/after survey:time since survey, before/after survey: flight day, and time since survey:flight day (details in Table D in S1 File). This top model of independent bear counts from hill camps indicated that, on average, there was a 62% decrease in bears within 100m of salmon streams concurrent with flights. In the control condition of days with no flights, there was only a 4% decrease across the time span when surveys ordinarily occurred. The dredge process for family groups (*n* = 423 detections) selected the model with only an intercept, indicating no effect from flights. The dredge process for males (*n* = 1381 detections) resulted in a top model similar to the top model for all bears, except without the time since survey:flight day interaction. The top model for males indicated that, on average, there was a 48% decrease in male bears within 100m of streams following survey flights. In the control condition of days with no flights, there was only a 18% decrease in males across the time span when surveys ordinarily occurred. We also found that the use of the autoregressive correlation structure (AR(1)) was justified for all models, because the estimated correlation between sequential observations after taking other predictors into account was large (ρ>0.97) for all models.

### Time-lapse camera data

**Fig 4:**
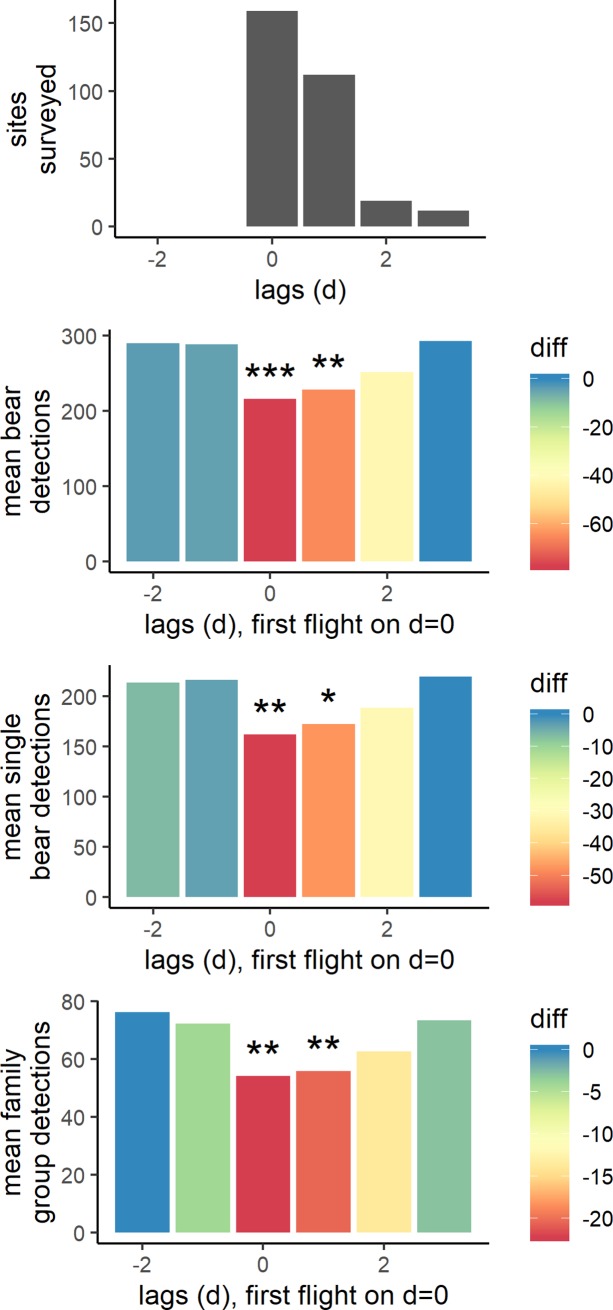
Composite of time-lapse detections of bears in the days before, during and after aerial surveys. The top panel shows the number of sites (salmon streams and rivers) surveyed in each composite lag. The first survey flight of each sequence occurred on lag = 0. The next three panels show detections of all bears, single bears, and family groups, respectively. Each bar shows the mean number of bears detected at each daily time lag, with aerial surveys occurring on day zero. The color shows the difference in number of detections from the lag with the highest mean bear detections. The bars show the composite of raw detections, however, bear presence at streams varies naturally throughout time due to factors such as changing salmon abundance and diel cycles. Thus, significance tests were performed on data with seasonal and daily patterns removed. We determined significance using a bootstrapping method where we compared periods with aerial surveys to randomly selected periods that did not have aerial surveys. P values show how much more extreme the aerial survey influenced observations were compared to non-aerial survey patterns. p<0.05, *; p<0.01, **; p<0.001, ***.

Time-series compositing removed both seasonal and diel variation from hourly time-lapse detections of bears near streams (Figure C in S1 File). Compositing of the remaining variation around the start of survey flights showed that daily bear detections decreased by 25.2% on the first day of aerial surveys (when cumulatively 159 sites were aerially surveyed) (Fig 4; p = 0.0005 with α = 0.05) and 21.0% the day after the start of surveys (when 112 sites were aerially surveyed) (p = 0.0075). Mean bear detections were 13.0% lower two days after the start of surveys when 19 sites were aerially surveyed (this difference was not statistically significant, p = 0.23). Mean bear detections appeared to return to pre-flight levels three days after a weekly round of surveys had started. The pattern of bear detections was very similar for single bears and families (sows with cubs), which had, respectively, 25.3% (p = 0.0007) and 25.1% decreases (p = 0.0017) on the day survey flights began and returned to pre-survey levels after three days.

### Location data from GPS collars

We identified 213 series of hourly GPS locations from collared female bears that overlapped aerial survey flights (24 hours before and after), had at least one location within 50m of streams, and included at least 30 locations after screening for accuracy. Results from our mixed effects model did not indicate a significant change in distance from survey streams following aerial survey flights (p = 0.924, Table E in S1 File). We also found that the use of the autoregressive correlation structure (AR(1)) was justified, because the estimated correlation between sequential distance observations after taking other predictors into account was high (ρ = 0.99).

## Discussion

Our results provide mixed support for the hypothesis that bears temporarily decreased activity near survey streams in response to aerial bear surveys. Evaluation of ground-based surveys on one stream revealed that most bears (62%) departed stream areas during and just following aerial surveys, but bear counts returned to pre-survey levels within three hours of flights. Our analysis of pairs of aerial surveys (*n* = 106) indicated that follow-up surveys counted 2% *more* bears than initial surveys, however, the average of 12.8 +/- 3.4 hours that passed between flights would have been too long to detect an effect as brief as that observed with ground surveys. While these two sources of data isolated the response of bears to single flight events, the time-lapse photo data allowed us to explore the cumulative response of bears to clusters of flights at ~12-hour intervals. These data showed that bear detections dropped by 25% on the first day of an aerial survey cluster and did not return to pre-survey levels for three days. This slow return to baseline bear numbers should not be interpreted as the length of time individual bears stayed away from a stream following the first survey flight. Rather, each survey flight briefly dispersed bears, but multiple flights in an aerial survey cluster cumulatively caused two to three days of reduced bear detections on streams.

Although we did see declines in bear use of salmon streams, it is unclear whether this negatively affected bear fitness. Recent research in the study area found a strong link between the number of days female bears spent fishing and their salmon consumption [35], and salmon consumption strongly predicts bear litter size and population density [36]. Thus, if the 25% decline in bear detections on the day of the survey resulted in 25% fewer salmon eaten, then this would almost certainly decrease bear fitness. However, while it is clear that bear consumption of salmon is sensitive to the number of days in a season that they can feed on salmon, it is less clear how sensitive they are to the number of hours in the day they can feed on salmon. When prey are abundant, predator foraging rates far exceed their rates of digestion (i.e., the digestive bottleneck), so energy intake is not limited by foraging time [37]. For example, captive bears fed *ad libitum* for 24 hours consume no more salmon than bears fed for 12 hours [38]. Where salmon runs are healthy, bears displaced from streams may simply return and feed to satiation later in the day. Bears have demonstrated behavioral plasticity when confronted with human-caused disturbance. For example, bears often shift their activities to avoid people, foraging or moving during night instead of day [29]. The bears in our study area could have used streams during nighttime to compensate. Unfortunately, we were unable to test the hypothesis that aerial surveys caused bears to shift the time and location of fishing activity. Overall, it is clear that bears responded to surveys by temporarily departing the vicinity of survey streams, but there is no clear evidence suggesting this reduces energy intake rates or other aspects of bear fitness.

The response of bears to aerial surveys appeared to be sex and class dependent. Ground observations indicated less displacement of female bears with cubs than males or single bears of unknown sex. Our GPS collar data did not show females moving away from aerial survey streams following flights. Unfortunately, we had no GPS data from males for comparison, so while we documented males being displaced we do not know how far they moved from streams. Our findings are consistent with prior evidence that male and female bears behave differently in places where hunting regulations target males [27]. Male bears in areas with selective hunting tend to more strongly shift to nocturnal activity patterns than females with cubs that are not hunted [29,39]. Compared to females in un-hunted populations, female bears in hunted populations tend to use areas nearer to humans [40] and carry litters longer (because they are not hunted while caring for cubs) [41]. From 2000 to 2013, the average hunting rate on the Kodiak Archipelago was ~8% of independent bears (excluding cubs), and was 2.8 times higher for males than females [42]. Thus, it is plausible that hunting explains the stronger male aversion to survey aircraft and other human activities.

In addition to the many examples of behavioral responses to aircraft (e.g. alertness and fleeing), there is evidence animals can experience stress responses without outward signs of fear. For example, a study of bears with implanted heart rate monitors and GPS collars revealed spikes in heart rates but not changes in movement rates during and immediately after overflights by UAVs [13]. Although it is not clear these stress responses decreased bear fitness, high stress has been linked to poor fitness in other animals [43]. These studies show that a lack of outward animal behaviors cannot alone be used to rule out negative effects from human activities. Finally, this study and others have focused on acute stress responses rather than the chronic stress that could occur in bears that are repeatedly exposed to aerial surveys. A future avenue for investigation may be to measure the stress hormones of bears exposed to varying amounts of human-caused disturbance to determine whether long-term exposure to aerial surveys cause chronic stress.

## Conclusions

This study demonstrated that aerial surveys, a key tool used for monitoring demographic composition and salmon stream use, may briefly displace bears from key foraging areas. One method detected a large magnitude of displacement—ground surveys documented 62% of bears departed one stream following survey flights. However, bears returned to pre-survey abundance within three hours of survey flights and analysis of the larger and more time-integrated time-lapse camera dataset detected a smaller, 25% reduction in bear detections. Flights did not seem to affect female bears with and without cubs, although our methods may not have been well-suited to detect differences in sex. The fitness of survey-displaced bears may be diminished where salmon fishing time [35] and salmon consumption are reduced [36]. However, bears are ecologically flexible and survey-displaced bears may simply adjust their pattern of daily salmon foraging activity to avoid aerial surveys. Local bear managers may wish to reconsider the efficacy of these surveys in their efforts to successfully manage bears. If the data from these surveys is valuable, then alternative methods such as remote wildlife cameras [44,45], Unmanned Aerial Vehicles (but see Ditmer et al. 2015), or high-altitude infrared aerial surveys [46,47] should be considered. However, potential new survey methods should be systematically assessed to ensure they are indeed lower impact. It is important to note we could not detect the (albeit short-term) decrease in bears at streams by analyzing the aerial survey counts. Instead, we needed independent sources of data with either higher temporal resolution (i.e. the ground surveys), or more time-integrated methods of measuring bear activity (i.e. time-lapse cameras). Based on this experience, we recommend others using or developing protocols for aerial surveys implement plans to independently monitor for negative effects. It is important that wildlife scientists and managers consider the potentially inadvertent negative impacts their efforts may have on the wildlife they are studying and managing. This is necessary for wildlife scientists and managers to avoid causing harm to the species they seek to study and manage.

## Supporting information

S1 File Supporting figures and tables. (DOCX)
