# Supplementary material for: Aerial surveys cause large but ephemeral decreases in bear presence at salmon streams in Kodiak, Alaska
Source: PLoS One. 2019 Sep 10;14(9):e0222085. doi: 10.1371/journal.pone.0222085 (PMC6736237; doi:10.1371/journal.pone.0222085)
Supplement: S1 File — (DOCX) [file pone.0222085.s001.docx]

**Supporting information**

Title: Aerial surveys cause large but ephemeral decreases in bear presence at salmon streams in Kodiak, Alaska

William W. Deacy^1^*, William B. Leacock^2^, Eric J. Ward^3^, and Jonathan B. Armstrong^1^

^1^ Department of Fisheries and Wildlife, Oregon State University, Corvallis, OR, USA.

^2^ Kodiak National Wildlife Refuge, United States Fish and Wildlife Service, Kodiak, AK, USA

^3^ Conservation Biology Division, Northwest Fisheries Science Center, National Marine Fisheries Service, National Oceanic and Atmospheric Administration, Seattle, WA 98112

^*^Corresponding author (will.deacy@gmail.com)

**Figure A.** **Diel patterns in bear behavior.** a) ground-based counts of bears at Connecticut Creek, Alaska; b) time-lapse camera detections (night time hours excluded because of poor detection ability; c) activity sensor data from GPS collars worn by 43 female bears. The red lines in all panels show the times of aerial survey flights.


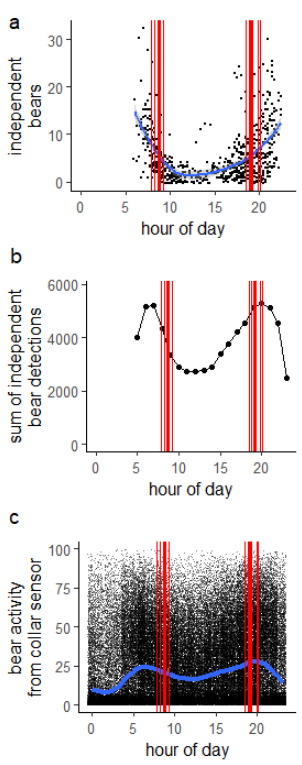


**Figure B. Bears observed from hill-camps above Connecticut Creek, Alaska.** Data has been divided between periods with aerial surveys (labeled flights) and no aerial surveys (labeled no flight). Points are jittered to alleviate over plotting and colored to indicate morning (red) or evening (blue) observations. The x axis shows the time relative to when surveys occurred (on flight days), or relative to the mean flight time (on non-flight days). Lines show LOESS fits. The top, middle, and bottom panels show data from single bears, family groups (a sow plus cubs counts as 1), and males bears, respectively.


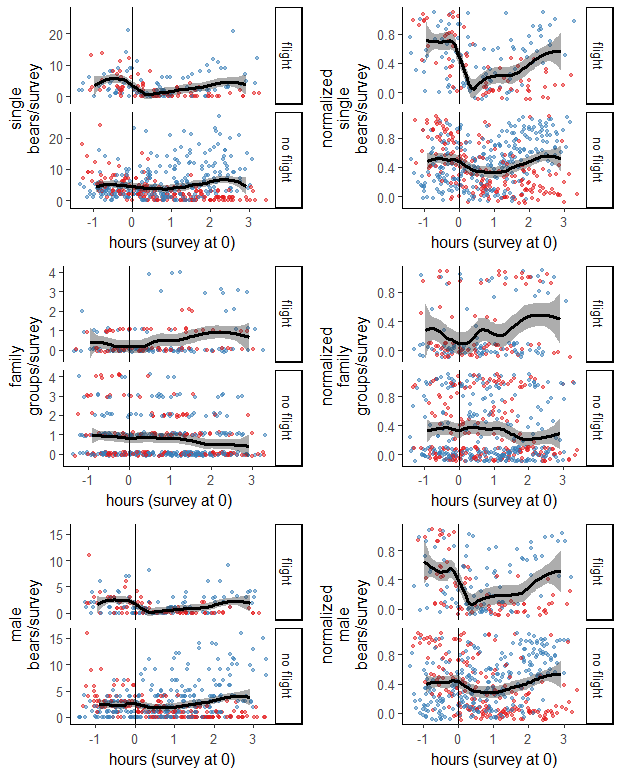


**Figure C**. **Decomposition of three years of bear detection data from time-lapse cameras deployed along ten streams in southwestern Kodiak, Alaska.** The top row shows raw hourly detections of bears summed across all sites, while the remaining show each step in the decomposition of hourly cam detections into seasonal, diel, and remainder components by LOESS. Using the remainder in analyses allows us to focus on responses to aerial surveys without these other sources of variation interfering. We used a seasonal (trend) LOESS window of 401 hours and a diel window of 25 hours. The bottom row shows the remainder after removal of seasonal and diel variation, with vertical red lines showing when aerial surveys occurred. We used the remainder in tests of bear response to aerial survey flights.


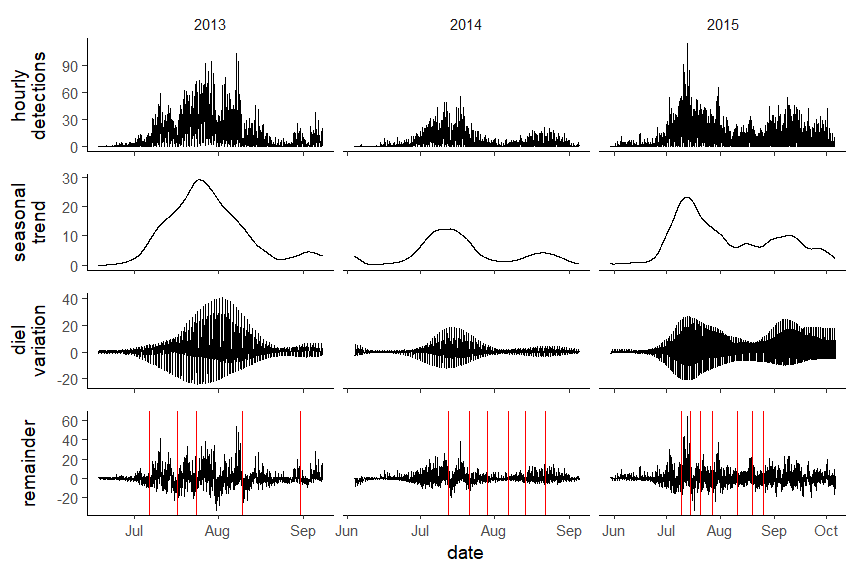


**Table A**. Fixed effects for model of brown bear stream survey counts as a function of survey sequence. The model included year and stream as random effects and a poisson error distribution. P values less than alpha (0.05) are in bold.

| FIXED EFFECTS | Estimate | Std. Error | z value | P value |
| --- | --- | --- | --- | --- |
| Intercept | 1.416328 | 0.318035 | 4.453 | **8.45E-06** |
| survey rep number | 0.029129 | 0.009235 | 3.154 | **0.00161** |

**Table B**. Fixed effects for model of difference in brown bear stream survey counts in paired sequential surveys as a function of time of day and hours since prior survey. Had year and stream as random effects and a gaussian error distribution.

| Fixed Effects | Estimate | Std. Error | t value | P value |
| --- | --- | --- | --- | --- |
| Intercept | -0.06596 | 0.240142 | -0.275 |  |
| Time (PM) | 0.330028 | 0.265664 | 1.242 | 0.214 |
| hours since last survey | 0.00486 | 0.009465 | 0.513 | 0.607 |

**Table C**. Top ten models from a dredge model selection process for ground survey counts of three bear classes. Colons indicate interactions between fixed effects.

| Bear Class | Rank | after flight | time (PM) | hours since | flight day (binary) | after: hours since | after: flight day | hours since: flight day | after: hours since: flight day | df | AICc | delta AICc | weight |
| --- | --- | --- | --- | --- | --- | --- | --- | --- | --- | --- | --- | --- | --- |
| All Bears | 1 | X | X | X | X | X | X | X |  | 11 | -3380.5 | 0.00 | 0.45 |
|  | 2 | X | X | X | X | X | X | X | X | 12 | -3378.4 | 2.08 | 0.16 |
|  | 3 | X | X | X | X | X | X |  |  | 10 | -3378.2 | 2.26 | 0.15 |
|  | 4 | X |  | X | X | X | X | X |  | 10 | -3377.8 | 2.67 | 0.12 |
|  | 5 | X |  | X | X | X | X | X | X | 11 | -3375.7 | 4.74 | 0.04 |
|  | 6 | X |  | X | X | X | X |  |  | 9 | -3375.6 | 4.86 | 0.04 |
|  | 7 | X | X | X | X |  | X | X |  | 10 | -3374.4 | 6.05 | 0.02 |
|  | 8 | X | X | X | X |  | X |  |  | 9 | -3372.3 | 8.16 | 0.01 |
|  | 9 | X | X |  | X |  | X |  |  | 8 | -3372 | 8.50 | 0.01 |
|  | 10 | X |  | X | X |  | X | X |  | 9 | -3371.3 | 9.20 | 0.01 |
|  |  |  |  |  |  |  |  |  |  |  |  |  |  |
| Family Groups | 1 |  |  |  |  |  |  |  |  | 4 | -12923.4 | 0.00 | 0.18 |
|  | 2 |  |  |  | X |  |  |  |  | 5 | -12922.4 | 1.00 | 0.11 |
|  | 3 |  |  | X | X |  |  | X |  | 7 | -12921.6 | 1.81 | 0.07 |
|  | 4 | X |  |  |  |  |  |  |  | 5 | -12921.4 | 1.92 | 0.07 |
|  | 5 |  |  | X |  |  |  |  |  | 5 | -12921.4 | 1.95 | 0.07 |
|  | 6 |  | X |  |  |  |  |  |  | 5 | -12921.4 | 1.99 | 0.07 |
|  | 7 | X |  |  | X |  |  |  |  | 6 | -12920.4 | 2.93 | 0.04 |
|  | 8 |  |  | X | X |  |  |  |  | 6 | -12920.4 | 2.96 | 0.04 |
|  | 9 |  | X |  | X |  |  |  |  | 6 | -12920.4 | 2.98 | 0.04 |
|  | 10 |  | X | X | X |  |  | X |  | 8 | -12919.6 | 3.82 | 0.03 |
|  |  |  |  |  |  |  |  |  |  |  |  |  |  |
| Male Bears | 1 | X | X | X | X | X | X |  |  | 10 | -6524.9 | 0.00 | 0.21 |
|  | 2 | X |  | X | X | X | X |  |  | 9 | -6524.3 | 0.55 | 0.16 |
|  | 3 | X | X | X | X | X | X | X |  | 11 | -6524.2 | 0.67 | 0.15 |
|  | 4 | X |  | X | X | X | X | X |  | 10 | -6523.6 | 1.25 | 0.11 |
|  | 5 | X | X |  | X |  | X |  |  | 8 | -6522.7 | 2.17 | 0.07 |
|  | 6 | X | X | X | X | X | X | X | X | 12 | -6522.6 | 2.30 | 0.07 |
|  | 7 | X | X | X | X |  | X |  |  | 9 | -6522 | 2.86 | 0.05 |
|  | 8 | X |  | X | X | X | X | X | X | 11 | -6522 | 2.89 | 0.05 |
|  | 9 | X |  |  | X |  | X |  |  | 7 | -6522 | 2.92 | 0.05 |
|  | 10 | X | X | X | X |  | X | X |  | 10 | -6521.3 | 3.58 | 0.04 |

**Table D**. Model parameters for models of ground counts of bears before and after aerial survey flights. There is a separate model for each demographic that was selected through a dredge process where all combinations of parameters are modelled, and then the model with the lowest AICc is selected as the top model. All models had AR(1) covariation and beta error distributions. P values less than alpha (0.05) are in bold.

|  |  | Estimate | Std. Error | z value | P(>\|z\|) |
| --- | --- | --- | --- | --- | --- |
| All Bears | intercept | -0.6067 | 0.4215 | -1.439 | 0.15006 |
|  | after flight (binary) | -0.1789 | 0.4318 | -0.414 | 0.67864 |
|  | time (PM) | 0.6553 | 0.2963 | 2.212 | **0.02699** |
|  | hours since flight | -1.5404 | 0.5841 | -2.637 | **0.00836** |
|  | flight day (binary) | 1.0683 | 0.4769 | 2.24 | **0.02508** |
|  | after flight: hours since flight | 1.7958 | 0.6284 | 2.858 | **0.00427** |
|  | after flight: flight day | -2.748 | 0.6685 | -4.11 | **3.95E-05** |
|  | hours since flight: flight day | 0.7173 | 0.3445 | 2.082 | **0.03732** |
|  |  |  |  |  |  |
| Family Groups | Intercept | -0.4846 | 0.09271 | -5.227 | **1.72E-07** |
|  |  |  |  |  |  |
| Males | Intercept | -0.85 | 0.3896 | -2.182 | **0.029129** |
|  | after flight (binary) | -0.5056 | 0.3356 | -1.506 | 0.132015 |
|  | time (PM) | 0.5129 | 0.3127 | 1.64 | 0.10092 |
|  | hours since flight | -0.7955 | 0.4592 | -1.732 | 0.083234 |
|  | flight day (binary) | 0.933 | 0.4495 | 2.076 | **0.037909** |
|  | after flight: hours since flight | 1.1105 | 0.5048 | 2.2 | **0.027812** |
|  | after flight: flight day | -1.6994 | 0.4576 | -3.714 | **0.000204** |

**Table E**. Fixed effects for model of change in linear distance (m) of GPS collared brown bears from salmon streams before and after aerial surveys. Had bear ID as a random intercept and a gaussian error distribution.

| Fixed Effects | Estimate | Std. Error | z value | Pr(>\|z\|) |
| --- | --- | --- | --- | --- |
| Intercept | 0.048724 | 0.082652 | 0.59 | 0.556 |
| After survey | 0.001391 | 0.014535 | 0.096 | 0.924 |
